# Survival of sharp force trauma in burnt bones: effects of environmental factors

**DOI:** 10.1007/s00414-022-02916-9

**Published:** 2022-11-23

**Authors:** Vijarn Vachirawongsakorn, Nicholas Márquez-Grant, Jonathan Painter

**Affiliations:** 1 Department of Forensic Medicine, Faculty of Medicine Siriraj Hospital, Mahidol University, Bangkok, Thailand; 2 Cranfield Forensic Institute, Cranfield University, Bedford, UK

**Keywords:** Taphonomy, Sharp force trauma, Forensic anthropology, Human remains, Burnt bone, Cut marks

## Abstract

This study investigates how environmental variables, such as temperature and rainfall, affect previously induced cut marks on burnt bones. This research used non-serrated and serrated blade knives to inflict trauma on *Sus scrofa* ribs (*n* = 240). The bones were later burnt and left for 1 month in a taphonomic experimental facility. Qualitative and quantitative examinations were conducted using macroscopic and microscopic techniques to assess specific characteristics of the cut marks. Any changes to the dimension and morphology of the cut marks as well as their level of fragmentation were recorded.

This study has led to three important outcomes: (1) identification of pre-existing cut marks is possible in reconstructed burnt bone fragments; (2) cut marks from different types of knife blades showed dissimilar responses to heat and the environment; and (3) specific environmental variables affect burnt bone fragmentation. These results have implications for trauma analysis on burnt remains in forensic anthropology casework.

## Introduction

In a previous paper [1], the characteristics of cut marks (marks made by the cutting action of a bladed weapon) on bone (pig ribs) caused by three different knives were assessed before and after burning. The study showed that the cut marks could be attributed to the different knife types (non-serrated, fine-serrated, and coarse-serrated) both before and after burning. However, alterations to the cut mark characteristics did result from burning, with the extent of the change dependent upon the characteristics of the cut mark and thus knife types. In this paper, the subsequent effect of exposure to the environment on the morphology of cut marks on burnt bones is investigated in order to further understand how cut marks may be modified as a result of taphonomic factors.

When dealing with burnt skeletal remains, bone tends to undergo a series of stages, resulting in both macro- and microstructural changes. These changes include dehydration, followed by pyrolysis of the organic component, inversion of the inorganic material, and fusion [2–5]. The end product is a calcined bone which has lost structural integrity and elasticity, making it vulnerable to fracture and fragmentation, the latter which can also be influenced by recovery and transportation processes [4]. However, it should be noted that the extent of both macro- and microstructural changes may differ due to the location, temperature, and duration of time during which the bone was exposed to heat.

Weathering refers to the compositional and physical breakdown of bone, as a result of exposure to the environment. Behrensmeyer (1978) postulated, in the first formal definition of bone weathering (of unburnt bone), that it is the process of separating and destroying the microscopic structure of the organic and inorganic components of bone [6]. This research and additional contributions [6–8] led to an index for classifying the effects of the deposition environment on the physical breakdown of bone. The potential effects of extrinsic and intrinsic variables should be taken into consideration when interpreting weathering stages [9]. Extrinsic variables include rainfall, temperature, and wind; while intrinsic variables include bone type, taxa, and size as well as pathological conditions such as osteoporosis. Certainly, the weakened structure of burnt bone undergoes differential weathering rates and patterns when compared with an unburnt bone [4, 9–11]. Significant improvements in this understanding have led to improved recovery of burnt skeletal remains in forensic cases [3, 12–15].

Therefore, the potential detrimental effect to cut mark characteristics that occurs when burning bone and any further degradation due to weathering needs further research. In this study, the burning was standardized by using a furnace set with a fixed time and temperature, so that a homogeneous environment could be provided as much as possible to better understand the effects of weathering.

## Materials and methods

To investigate the effect of environmental factors, 304 *Sus scrofa* (domestic pig) ribs were prepared. Full details of the methodology for rib preparation, inflicting cut marks, and burning are given in Vachirawongsakorn et al. (2022) [1]. Rib bones were chosen to simulate a stab to the chest scenario. Ribs measuring approximately 25 cm in length and 7 cm in width were dissected from racks of side ribs, the size chosen for consistency. The side ribs from juvenile domestic pigs were obtained from a local butcher shop. The majority of adherent muscle, tendon, and periosteum was removed by scissors and forceps ensuring no tool contact with the rib surface. The number of ribs chosen was to ensure that they could be subdivided into four seasonal groups (spring, summer, autumn, winter). For each season, 60 ribs had cut marks inflicted (3 cuts per bone) and were later burnt in a conventional furnace at a temperature of 850 °C for 30 min. A total of 16 control samples with no sharp force injury were also burnt and used as a baseline to investigate the effect of the environment. In total, 720 cut marks on 240 ribs, in addition to 64 control ribs, were employed in the study. While these conditions are not necessarily the same as a real burning/cremation scenario, the aim of this study was to investigate changes under controlled conditions. Further work replicating real fire scenarios is needed in future studies.

Three types of common kitchen knives were used: one with a non-serrated edge, a second one with a fine-serrated edge, and a third one with a coarse-serrated edge (see [1]). During the cutting procedure, the respective knife was moved with a single back and forth motion perpendicular to the longitudinal axis of the bone surface. Four identical knives of each type were purchased, with each knife used on 60 ribs (one season set) before being replaced to minimize the effect of a damaged blade.

### Analytical methods

To assess changes to the trauma morphology, each cut mark was characterized both with the naked eye and by stereomicroscopic examination to determine qualitative and quantitative characteristics of its morphology (Table 1). This was undertaken before and after burning and then at specific time intervals throughout the environmental exposure as detailed below. Bone surface modifications were also examined to evaluate overall taphonomic changes. The limitations of direct assessment of the cut marks by stereomicroscope observation, i.e., the inability to identify the finest surface detail, are acknowledged. Ideally the cut marks would be cast [15, 16]; however, it was considered possible that repeated casting of the cut marks could compromise this study as the likely degradation due to burning and then by environmental exposure were unknown.

**Table 1:** Morphological variables and analytical processes; E, naked eye examination; M, microscopic examination

Variables	Method		Definition	Findings and their definitions
	E	M		
Kerf length	X		Maximum distance between the starting and ending point of a kerf	-
Kerf width		X	Maximum distance between the outermost margins of a kerf	-
Kerf shape	X	X	Overall top-view shape of a kerf	Linear: narrow and parallel kerf margins and walls Elliptical: two inward-angled kerf margins that end at their tips with the broadest distance at the middle of the kerf Rectangular: parallel kerf margins and walls that end at their small U-shaped tips Irregular: irregularity of kerf morphology that cannot be categorized into any types of kerf shape
Kerf margin	X	X	Marginal morphology of a kerf	Smooth: a regular, smooth, and flat kerf margins Raised: an uneven, lateral raised margin of a kerf that attaches to the bone
Cross-sectional view		X	Cross-sectional shape of a kerf	V-shaped: two inward-angled kerf walls that end at the floor with the widest distance at the margin and narrower when the kerf walls descend to their floor U-shaped: two parallel walls that are connected by a curved floor with an equal distance between the walls Narrow: two parallel walls with a particularly narrow distance between the kerf walls and margins
Striations		X	Parallel striations on a kerf wall	Present, absent

Thermal fracture(s) of the bones were categorized and documented for every rib. When heat-induced fractures intersected a cut mark, the cut mark was recorded but omitted from the study. These, however, accounted for a small proportion of the cut marks (5.8%), and since there were undamaged cut marks on the same ribs, those ribs were still used in the study. Every burnt bone fragment was measured (length and breadth) using a digital caliper. Each fragment was then sorted into one of the three defined categories based on its smallest dimension (Table 2). Additionally, the mass of every fragment was weighed before and after the burning process with an Ohaus Adventurer® analytical electronic balance.

**Table 2:** Definition of the fragmented size of a burnt bone

Category	Definition
Small category	A bone fragment with a maximum dimension smaller than 1 mm
Medium category	A bone fragment with the maximum dimension between 1 and 5 mm
Large category	A bone fragment with a dimension larger than 5 mm

### Field observations

The experiments were started in the seasonal times of spring (May), summer (August), autumn (November), and winter (February) in the F3 taphonomic facility at Shrivenham campus of Cranfield University, Watchfield, Oxfordshire, UK. This area is a rural area of the Cfc climate of the Köppen-Geiger climate classification system (described as a warm temperate minimum temperature between – 3 °C and 18 °C, and the lowest precipitation of the hottest month being greater than 40 mm) [17]. Fundamental weather data including temperature, precipitation, wind speed, and sunshine hours was collected daily from the weather station near Brize Norton, West Oxfordshire (approximately 17 km away).

In order to prevent animal scavenging, the F3 taphonomic facility was enclosed by chain-link wires and wooden plank fences. For each season, 76 burnt bones were deposited in the facility: 60 traumatized and 16 control ribs. Of those, 40 traumatized and 8 control ribs were deposited on the surface, while the remaining 20 traumatized and 8 control ribs were buried at a depth of 60 cm. Each surface rib was located separately from each other by placing it at the center area of a 1 × 1-m rope grid. On a weekly basis, all surface samples were examined in situ to record general observations and traumatic morphological changes as well as the degree of fragmentation. In addition, 10 traumatized and 2 control ribs were recovered and taken to the laboratory to enable more detailed analysis. For the buried bones, on a bi-weekly basis, 10 traumatized and 4 control ribs were carefully recovered (along with any fragments) and examined in the laboratory. After cleaning the ribs with a soft brush, the analysis of both the surface and buried ribs comprised a morphological assessment, as well as dimensional and mass measurements of the ribs and of any fragments resulting from weathering. After the laboratory investigation, the ribs were removed from the study. In sum, after a period of 4 weeks, all the samples were retrieved from the facility and brought to the laboratory for further analysis.

In addition, the soil pH and moisture content were monitored for the buried bones. Around 10 g of soil was collected from the base of each burial pit at both the start and end of the 4-week experimental period. Approximately 5 g of soil samples was diluted and tested with a pH meter to measure soil pH, while the same sample was heated in an oven at 105 °C for 24 h to vaporize water, so that % of moisture content could be calculated [18].

## Results

### Weather and soil condition during field experiments

**Fig. 1:**
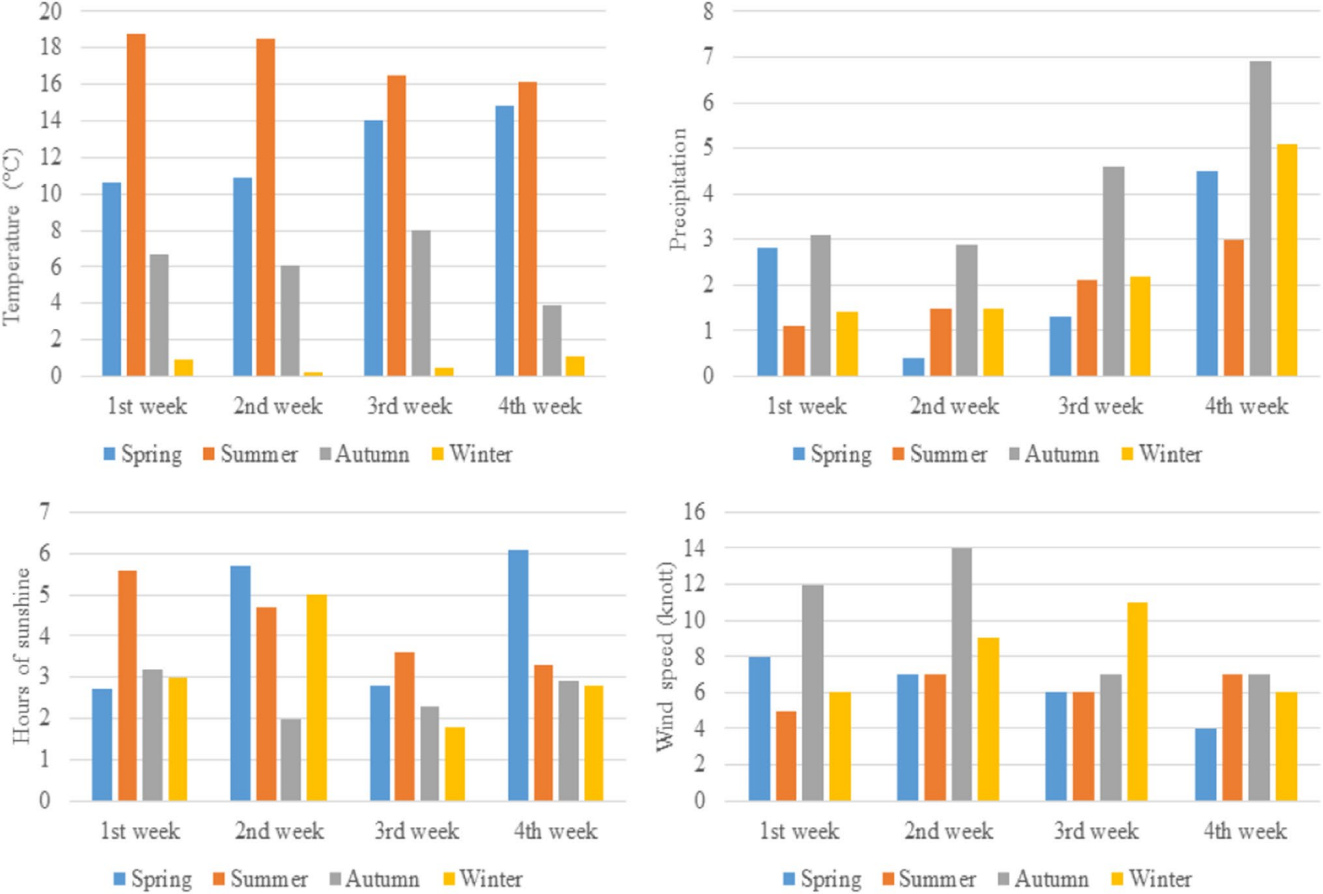
Summary of average weather condition in the spring (May 2017), summer (August 2017), autumn (November 2017), and winter (February 2018)

The average weekly weather condition for the F3 taphonomic facility during the months in which the samples were placed are shown in Fig. 1. The highest temperature with the longest period of daily sunshine in Southeast England was experienced during summertime, while significant rainfall with strong winds was observed in the autumn. The winter period was the only time where temperatures fell below freezing and with strong wind speeds.

Soil analytical data, namely pH and moisture, were documented at the start and at the end of each season (Table 3). The soil was consistently mildly acidic with pH values ranging between 5.89 and 6.51. A decrease in soil moisture was detected during the summer, whereas for all the other seasons the moisture content increased, with the highest increase occurring in the autumn. These findings correspond with the rainfall data.

**Table 3:** Soil data at the start and at the end of the field experiment

Season	Soil pH		Soil moisture (%)	
	Start	End	Start	End
Spring	6.43	6.51	24.92	25.14
Summer	6.15	6.03	21.73	21.44
Autumn	5.89	5.93	24.94	25.22
Winter	6.08	6.11	24.65	24.71

### Pre- and post-burnt examination

The data regarding the dimensional and morphological changes following burning are fully outlined in Vachirawongsakorn et al. (2022) [1]. To summarize, despite the cut marks from the different types of knives responding differently to burning, all the assessed cut marks in the study were macroscopically identifiable and could still be associated with the type of knife. The cut marks from the different knife blades showed a statistically significant reduction in kerf width, with a decrease between 28.5 and 34.9%. Additionally, changes to the kerf shape of cut marks from the fine- and coarse-serrated blades, as well as the kerf margin from the coarse-serrated blade, were also statistically significant. In contrast, only a small proportion (4.6%) of cut marks inflicted by the non-serrated blade underwent a shape change. Therefore, the changes to the cut marks resulting from burning did not alter the overall ability to distinguish cut marks inflicted by non-serrated blade from those inflicted by serrated blades.

### Post-environmental exposure

#### Bone fragmentation

**Fig. 2:**
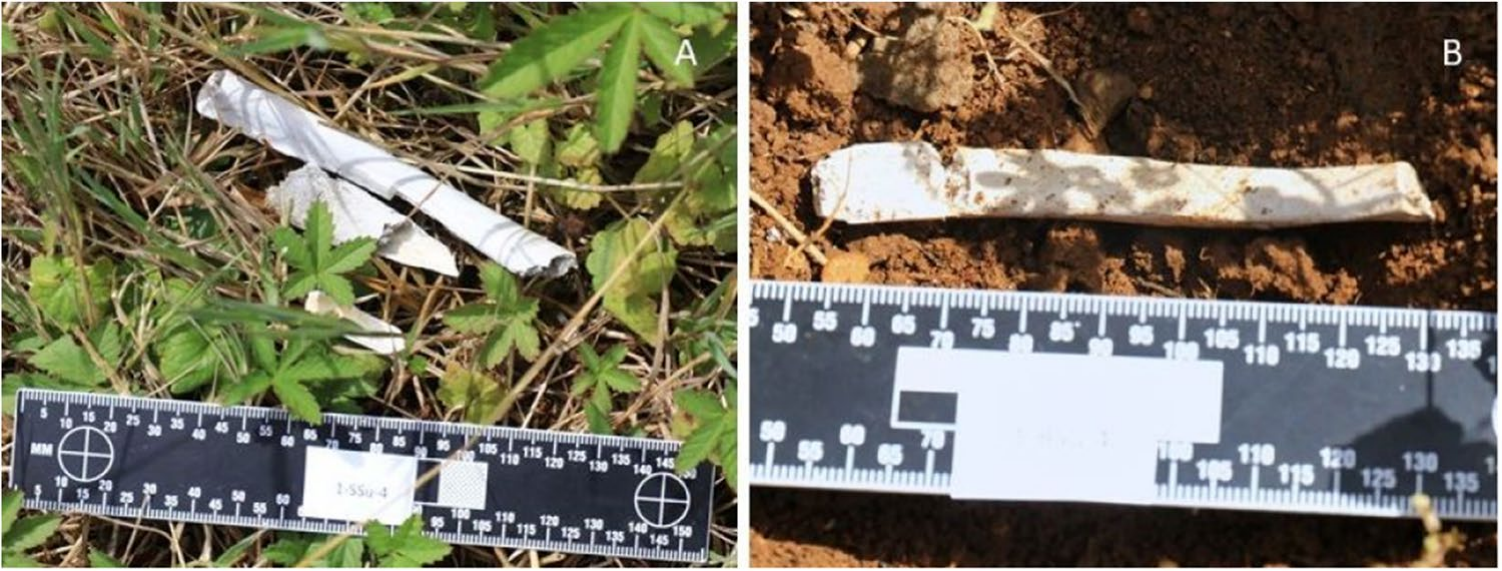
More fragmentation was observed in surface-deposited samples (**A**) than buried samples (**B**)

**Fig. 3:**
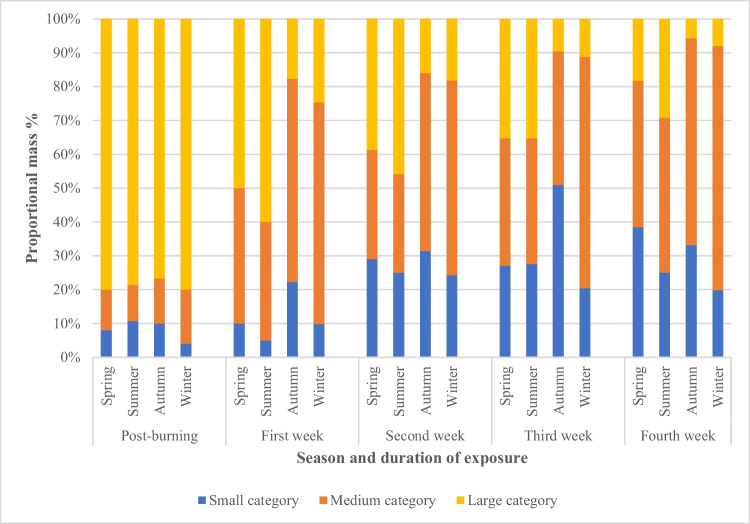
Percentage of proportional mass distribution of fragmented surface-deposited samples after the burning process and post-environmental exposure

**Fig. 4:**
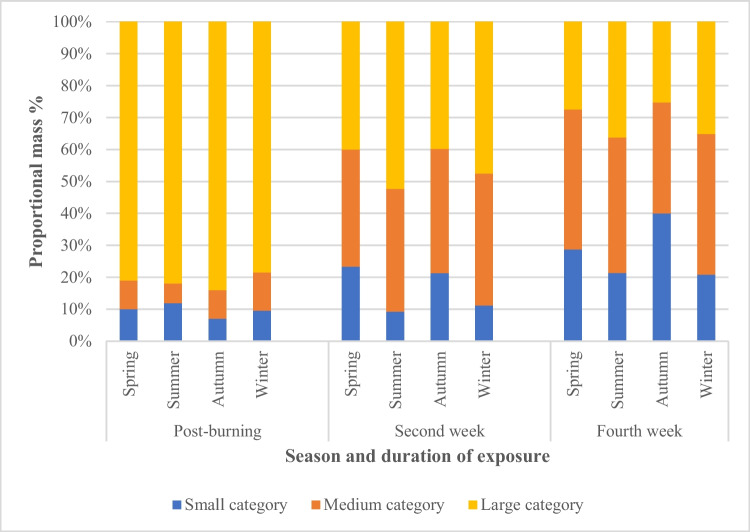
Percentage of proportional mass distribution of fragmented buried samples after the burning process and post-environmental exposure

After the full 4-week environmental exposure, all the burnt bones had undergone some degree of fragmentation, with the surface deposited bones undergoing more fragmentation compared to the buried bones (Fig. 2). In addition, the number of fragmented pieces was observed to gradually increase over time. Observations revealed that most of the fragmentation was caused by an extension from pre-existing heat-induced fractures. In addition, it was noticed that there was no clear difference between the fragmentation characteristics of the control and traumatic burnt groups; i.e., cut marks did not noticeably exacerbate fragmentation. To further categorize the degree of fragmentation, the mass of every recovered fragment was measured and sorted into one of three defined size groups: small, medium, and large (as defined earlier in Table 2). Figures 3 and 4 plot the proportion of the fragment size groups as a fraction of the whole for the surface and buried ribs, respectively. In both figures, the post-burning data refers to the fragment distribution immediately prior to starting the environmental exposure for that season.

For the surface deposited bones, after 1 week of exposure, the highest proportional increase for all seasons was in the medium category, with the autumn/winter samples undergoing higher fragmentation. The noticeable difference between the spring and autumn fragmentation is of particular interest as the average rainfall levels were similar, but the autumn bones underwent the highest level of fragmentation to both medium and small categories.

After the second week of surface exposure, all seasonal groups showed a substantial increase in the small-sized category. In particular, the spring and summer groups saw transitions from large-sized to small, whereas in the winter, there was a greater degree of further fragmentation of the medium-sized fragments. In the third week of exposure, further fragmentation of the large-sized category was observed. The summer group showed the highest rate of fragmentation, with the large-sized category reduced by 10%. Also, a substantial increase in the small-sized category in the autumn group was observed. During the fourth week of exposure, the spring group experienced the highest fragmented rate as can be seen from a large decrease of 17% in the large-sized category compared to the previous week and a marked increase in the amount of medium- and small-sized categories. Almost all large-sized categories of the autumn and winter groups were eliminated, with the autumn group showing a higher fragmentation rate than the winter group. The summer group showed the best survival rate with the highest proportion of larger fragments of nearly 30%.

As mentioned earlier, the hand excavation and the recovery of buried burnt bones were carried out after 2 weeks to enable analysis of the level of fragmentation (as well as cut mark assessment). The proportions of the fragmentation sizes, as shown in Fig. 4, vary between the seasons. However, similarities in the patterns of fragmentation are apparent between the summer and winter groups and to a lesser degree between the spring and autumn groups. The latter groups showed a higher fragmentation rate resulting in a smaller percentage of the large-sized category compared with the summer and winter groups. The same pattern of fragmentation rate continued into the fourth week. However, the highest overall level of fragmentation was observed in the autumn group. Conversely, the summer and winter samples had similar but lower overall levels of fragmentation. Nevertheless, better preservation of burnt bone fragments in all seasonal groups was detected compared with the surface-deposited group (Fig. 3).

#### Loss of burnt bone weight

**Fig. 5:**
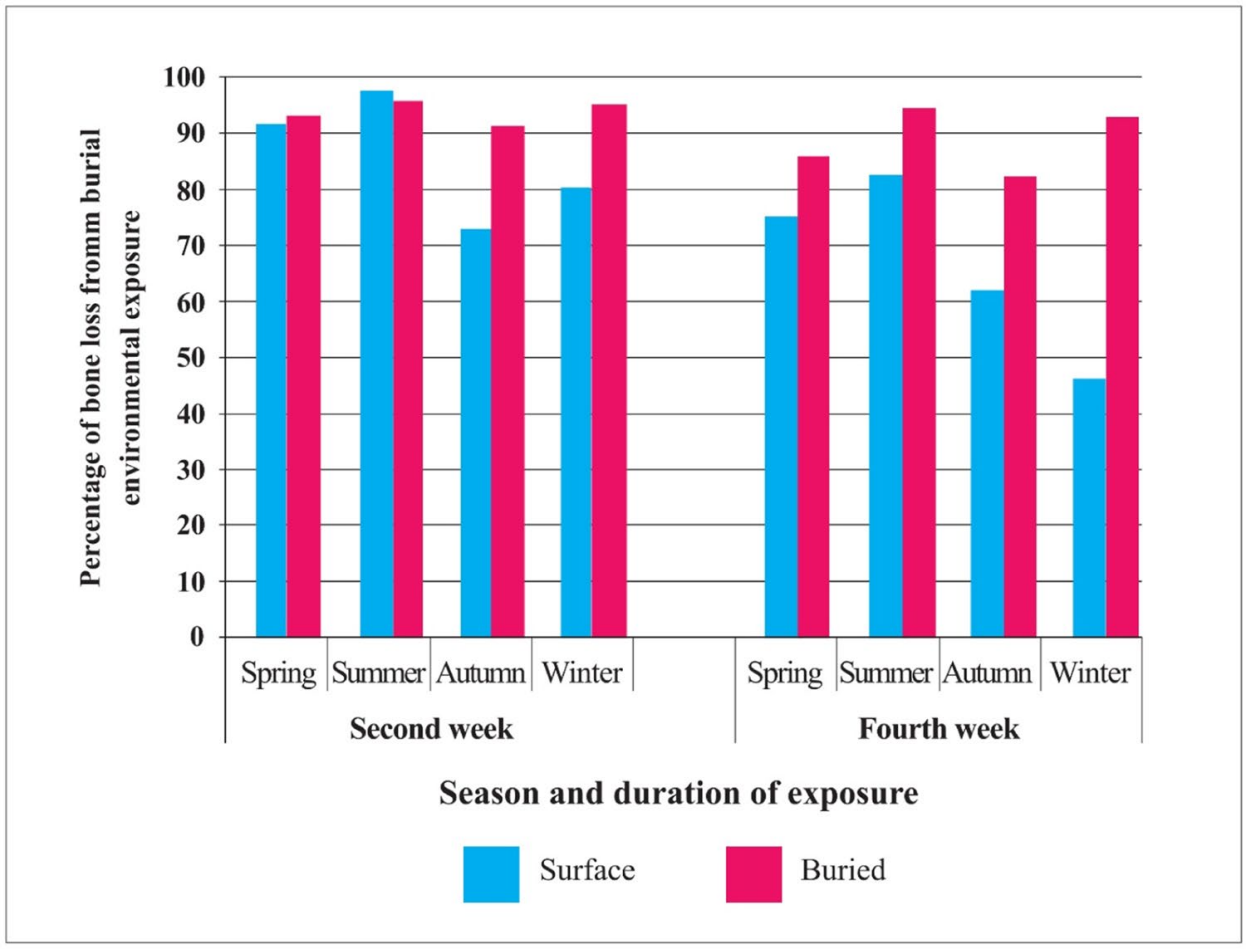
Percentage of weight loss of surface and buried burnt bones and according to each season

The weight of each burnt rib was measured before and after burning, and then after 2- and 4-week exposure to investigate the effect of a specific environment on the survival, and therefore recoverability of burnt bone. The results of burnt bone weight are presented in Table 4 and Fig. 5. The percentage of residual bone weight after environmental exposure was calculated by dividing the post-environmental exposure weight by the post-burnt weight to account for initial bone mass variation. The weight loss is therefore attributed to unrecovered fragmentation (i.e., fragments so small that they are not readily observable within the soil on recovery) of the bones.

**Table 4:** Average weight and standard deviation of burnt bone weight and percentage of residual weight after heat exposure, surface (S), and buried (B) environmental exposure

Season and exposure time			Pre-burn (gram)	Post-burn (gram)	%Residual weight after burn	Post-exposure (gram)	%Residual weight after exposure*
Spring	2wk	S	12.99 ± 2.95	2.99 ± 0.62	23.142	2.73 ± 0.57	91.432
		B	12.13 ± 1.33	2.72 ± 0.41	22.416	2.53 ± 0.4	92.878
	4wk	S	13.33 ± 2.1	3.25 ± 0.57	24.356	2.44 ± 0.55	75.08
		B	14.18 ± 2.32	3.35 ± 0.56	23.666	2.86 ± 0.57	85.348
Summer	2wk	S	13.11 ± 2.62	3.48 ± 0.63	26.618	3.38 ± 0.63	97.182
		B	13.9 ± 3.6	3.8 ± 1.05	27.226	3.61 ± 0.91	95.118
	4wk	S	13.45 ± 3.48	3.89 ± 1.02	28.874	3.21 ± 0.77	82.434
		B	11.85 ± 2.51	3.22 ± 0.62	27.524	2.97 ± 0.56	92.232
Autumn	2wk	S	12.39 ± 1.67	3.22 ± 0.48	26.01	2.36 ± 0.48	73.244
		B	12.45 ± 3.58	2.83 ± 0.87	22.686	2.55 ± 0.82	90.174
	4wk	S	15.33 ± 2.76	3.61 ± 0.65	22.35	2.24 ± 0.54	62.17
		B	12.55 ± 2.11	3.02 ± 0.68	23.858	2.48 ± 0.61	82
Winter	2wk	S	12.78 ± 2.94	3.09 ± 0.62	24.288	2.47 ± 0.58	80.092
		B	12.93 ± 3.12	3.17 ± 0.78	24.576	3 ± 0.62	94.546
	4wk	S	14.12 ± 2.39	3.37 ± 0.76	23.74	1.56 ± 0.47	46.4
		B	13.43 ± 2.73	3.32 ± 0.67	24.776	3 ± 0.74	90.416

*Comparison between pre-exposure and post-exposure weight

Due to the natural variation in rib sizes, there was a pre- and post-burning weight variation. The post-burn residual weight varied between 22 and 29% of the fresh bone weight. However, it is considered that the duration in the furnace was sufficient to cause the loss of all water and organic material from the ribs. The weight loss following environmental exposure (i.e., unrecovered bone) displayed similar characteristics to the fragmentation in the previous section. For the surface exposure samples, the autumn group demonstrated the poorest survival after the first 2 weeks, while the winter group showed the lowest survival rate after 4 weeks. In contrast, the summer group exhibited the highest survival rate when compared with the other seasons. In comparison, buried burnt bone displayed a better overall survival rate for all the seasons. The lowest survival rate was during the autumn, followed by the spring group.

#### Cut mark analysis

**Fig. 6:**
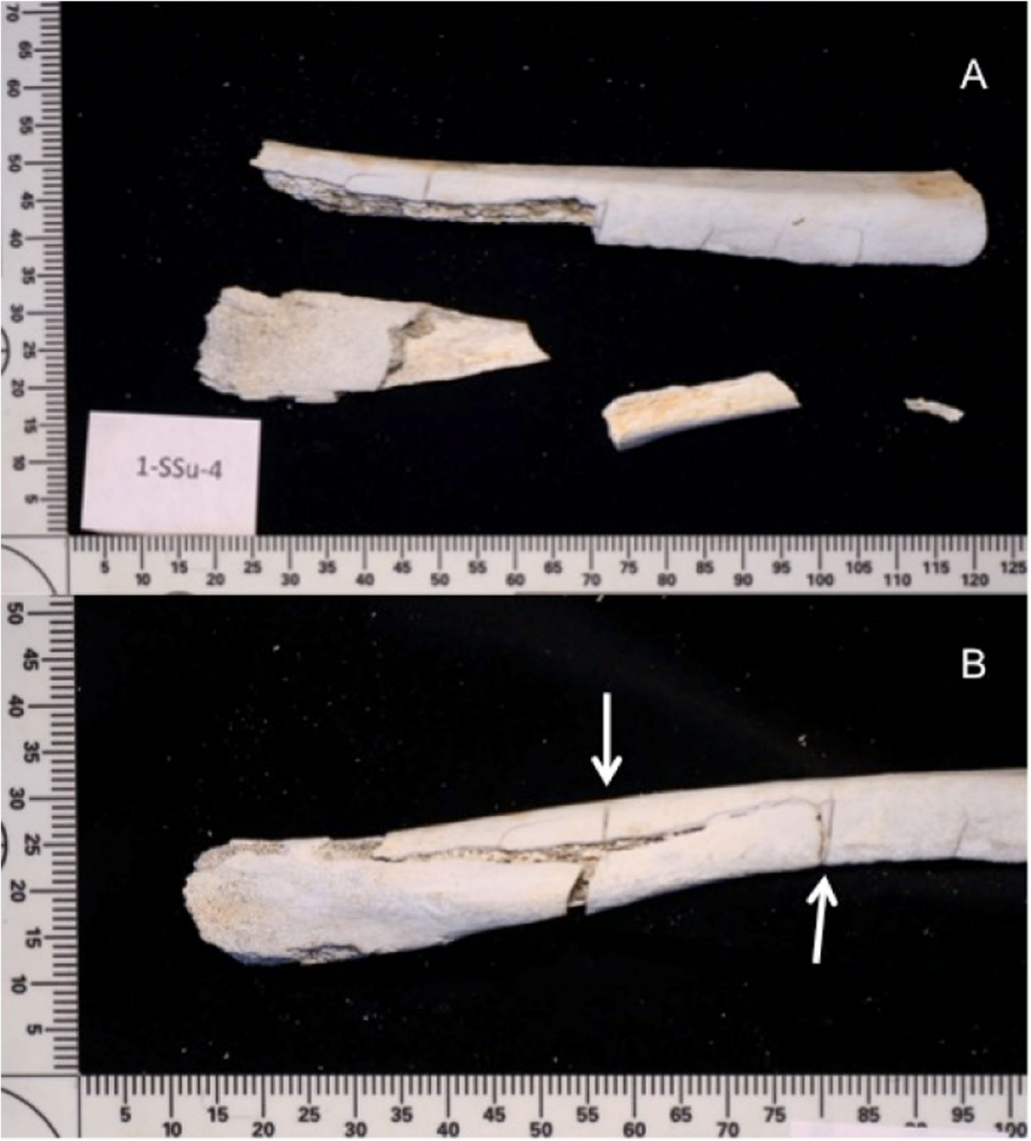
A surface exposure cremated rib before (**A**) and after (**B**) reconstruction; the white arrows indicate reconstructed cut marks

After environmental exposure, it was possible to assess the majority (72.2%) of cut marks, i.e., the cut marks that were still identifiable and measurable. However, there were differences in preservation rates, particularly between the surface samples. A cut mark was deemed to be available for full analysis if heat-induced fracturing of the bone did not affect the cut mark. Intact cut marks with clearly defined morphology were analyzed. If the fracture intersected a cut mark, the dimensional and morphological characteristics could not be reliably assessed and were omitted from the analysis. In some cases, the high fragmentation of ribs required reconstruction with glue to enable examination of the cut marks and their correlated defects (Fig. 6), but this was only to enable the spatial relationships of the cut marks/defects with the rib.

**Fig. 7:**
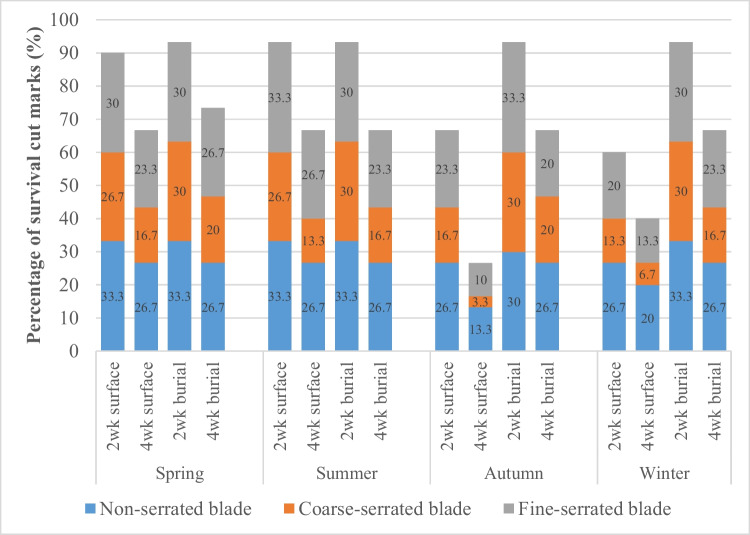
Incidence of survival cut marks comparing between each season

Figure 7 illustrates the presence of cut marks that have survived or not survived after environmental exposure irrespective of knife type. As shown, after 4-week exposure, the percentage of cut marks on buried ribs that were still observable is fairly constant for all seasons (66–73%), more so for the spring and summer surface samples (66–68%). In contrast, the level of destruction of cut marks increased markedly in the autumn and winter surface samples. The most damaging period occurred during the autumn as only 26.7% of cut marks survived after 4 weeks. Interestingly the biggest declines in any 2-week period were during the second 2 weeks in the autumn trial (40%) and the first 2 weeks of winter (40%). Note these were not consecutive weeks.

On closer inspection, changes to the dimensions and morphology of all the cut marks were observed after surface environmental exposure. Both the length and width of cut marks are altered compared with pre-exposure measurements. There was a negative correlation between the kerf length and time of exposure in all seasons, but there was a smaller reduction of the kerf length in the summer group. In addition, approximately, 80% of the cut marks showed a negative correlation between their kerf width and time of exposure. However, there was an increase in kerf width in the 2-week surface-deposited autumn and winter samples inflicted by coarse-serrated and fine-serrated blade knives and the 4-week surface-deposited winter samples inflicted by fine-serrated blades. A majority (83.3–96.7%) of 4-week surface-exposed cut marks inflicted by a coarse-serrated blade were badly damaged and could not be analyzed in every season. Nonetheless, a non-significant change of kerf dimensions was detected in all seasons (*p* > 0.05).

**Fig. 8:**
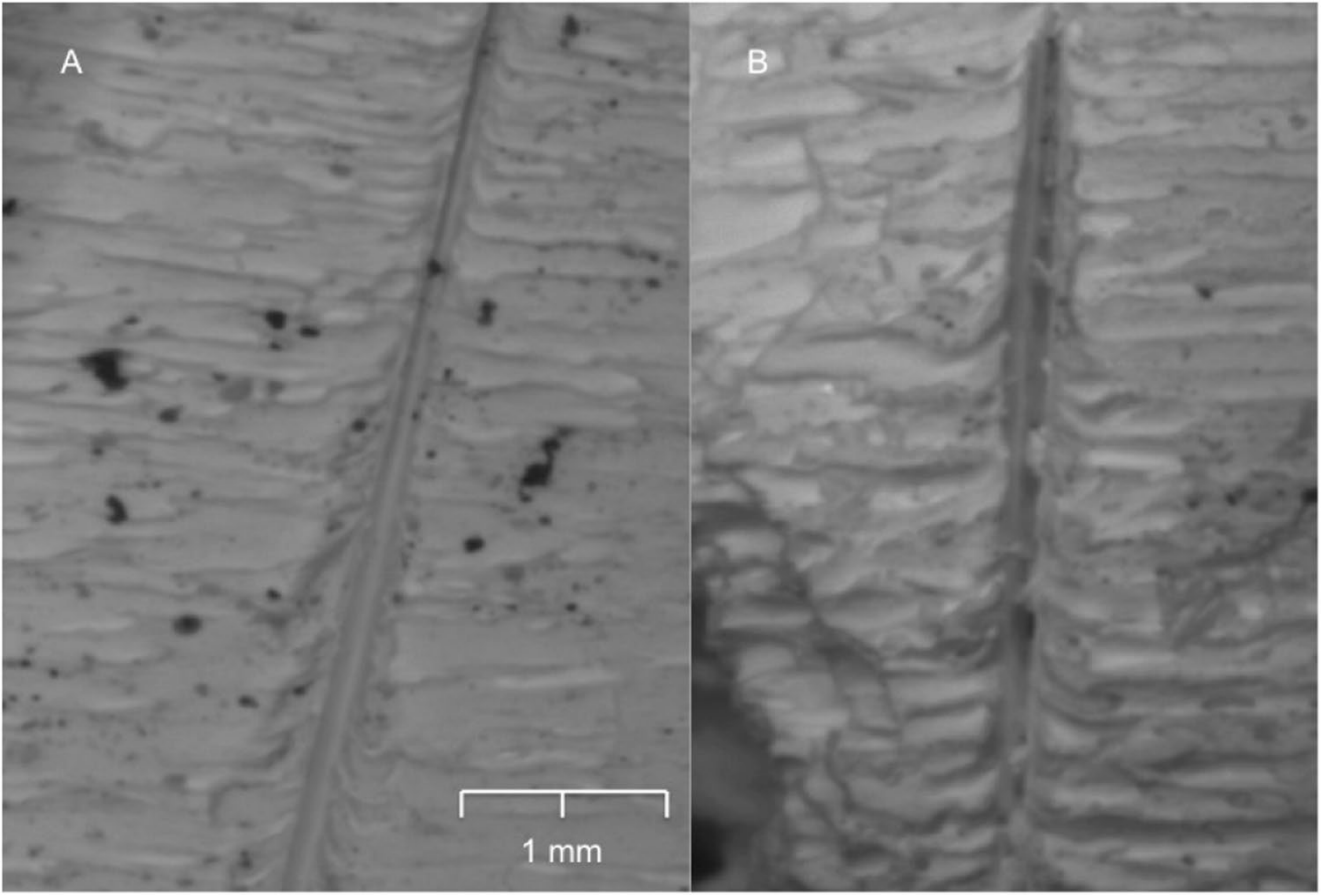
A small modification of kerf shape morphology from a non-serrated blade changed from a linear (**A**) to an elliptical shape after a 4-week environmental exposure (**B**)

It is clear that the environmental exposure, as well as the variation in the inflicted damage to the bone due to the different knife blade types, played an important role in the degree of modification of each cut mark (Fig. 7, and Tables 5 and 6). For the non-serrated blade, morphological changes were observed but only in a small subset of surface exposed samples. The stable features for all the cut marks were both a smooth kerf margin and kerf wall (i.e., no striations), both features indicating a non-serrating blade. However, there was a slight change in the kerf shape from linear to elliptical; the transformation occurred in 25% of the winter group and 12% of those in the autumn group (Fig. 8). Nonetheless, there was no statistical significance in the change to the surface groups. No changes were detected in the buried sample.

**Table 5:** Summary of kerf morphological changes between pre-exposure, 2-week surface exposure (2wk E), and 4-week surface exposure (4wk E) cut marks from a non-serrated knife (NS), a coarse-serrated knife (CS), and fine-serrated knife (FS)

		Spring		Summer		Autumn		Winter	
		2wk E	4wk E	2wk E	4wk E	2wk E	4wk E	2wk E	4wk E
Kerf shape	NS	Unchanged	Unchanged	Unchanged	Unchanged	Unchanged	12.5% linear to ellipse	Unchanged	25% linear to elliptical
	CS	14.3% ellipse to irregular	25% ellipse to irregular	14.3% ellipse to irregular	33.3% ellipse to irregular	42.8% ellipse to irregular	75% ellipse to irregular	25% ellipse to irregular	57.1% ellipse to irregular
	FS	12.5% ellipse to irregular	25% ellipse to irregular	11.1% ellipse to irregular	22.2% ellipse to irregular	25% ellipse to irregular	33.3% ellipse to irregular	11.1% ellipse to irregular	37.5% ellipse to irregular
Cross-sectional shape	NS	Unchanged	Unchanged	Unchanged	Unchanged	Unchanged	Unchanged	Unchanged	Unchanged
	CS	Unchanged	Unchanged	Unchanged	16.7% V to U	Unchanged	33.3% V to U	Unchanged	33.3% V to U
	FS	12.5% V to U	11.1% V to U	12.5% V to U	Unchanged	22.2% V to U	25% V to U	22.2% V to U	37.5% V to U
Kerf margin	NS	Unchanged	Unchanged	Unchanged	Unchanged	Unchanged	Unchanged	Unchanged	Unchanged
	CS	16.7% raised to smooth	33.3% raised to smooth	14.3% raised to smooth	33.3% raised to smooth	50% raised to smooth	85.7% raised to smooth	33.3% raised to smooth	71.4% raised to smooth
	FS	25% raised to smooth	33.3% raised to smooth	Unchanged	25% raised to smooth	33.3% raised to smooth	60% raised to smooth	50% raised to smooth	66.7% raised to smooth
Kerf striations	NS	Unchanged	Unchanged	Unchanged	Unchanged	Unchanged	Unchanged	Unchanged	Unchanged
	CS	14.3% presence to absence	16.7% presence to absence	Unchanged	28.6% presence to absence	28.6% presence to absence	42.9% presence to absence	14.3% presence to absence	33.3% presence to absence
	FS	Unchanged	16.7% presence to absence	14.3% presence to absence	28.6% presence to absence	16.7% presence to absence	50% presence to absence	28.6% presence to absence	33.3% presence to absence

Shading highlights statistical significance

**Table 6:** Summary of kerf morphological changes between pre-exposure, 2-week burial exposure (2wk E), and 4-week burial exposure (4wk E) cut marks from a non-serrated knife (NS), a coarse-serrated knife (CS), and fine-serrated knife (FS)

		Spring		Summer		Autumn		Winter	
		2wk E	4wk E	2wk E	4wk E	2wk E	4wk E	2wk E	4wk E
Kerf shape	NS	Unchanged	Unchanged	Unchanged	Unchanged	Unchanged	Unchanged	Unchanged	Unchanged
	CS	Unchanged	14.3% ellipse to irregular	Unchanged	28.6% ellipse to irregular	12.5% ellipse to irregular	42.9% ellipse to irregular	16.7% ellipse to irregular	22.2% ellipse to irregular
	FS	11.1% ellipse to irregular	25% ellipse to irregular	Unchanged	11.1% ellipse to irregular	12.5% ellipse to irregular	37.5% ellipse to irregular	Unchanged	33.3% ellipse to irregular
Cross-sectional shape	NS	Unchanged	Unchanged	Unchanged	Unchanged	Unchanged	Unchanged	Unchanged	Unchanged
	CS	Unchanged	Unchanged	Unchanged	Unchanged	Unchanged	16.7% V to U	16.7% V to U	16.7% V to U
	FS	11.1% V to U	25% V to U	Unchanged	25% V to U	12.5% V to U	37.5% V to U	11.1% V to U	33.3% V to U
Kerf margin	NS	Unchanged	Unchanged	Unchanged	Unchanged	Unchanged	Unchanged	Unchanged	Unchanged
	CS	14.3% raised to smooth	33.3% raised to smooth	14.3% raised to smooth	16.7% raised to smooth	33.3% raised to smooth	42.9% raised to smooth	16.7% raised to smooth	28.6% raised to smooth
	FS	Unchanged	33.3% raised to smooth	Unchanged	25% raised to smooth	Unchanged	66.7% raised to smooth	25% V to U	66.7% raised to smooth
Kerf striations	NS	Unchanged	Unchanged	Unchanged	Unchanged	Unchanged	Unchanged	Unchanged	Unchanged
	CS	Unchanged	Unchanged	Unchanged	Unchanged	16.7% presence to absence	33.3% presence to absence	14.3% presence to absence	33.3% presence to absence
	FS	Unchanged	16.7% presence to absence	Unchanged	14.3% presence to absence	14.3% presence to absence	28.6% presence to absence	Unchanged	33.3% presence to absence

**Fig. 9:**
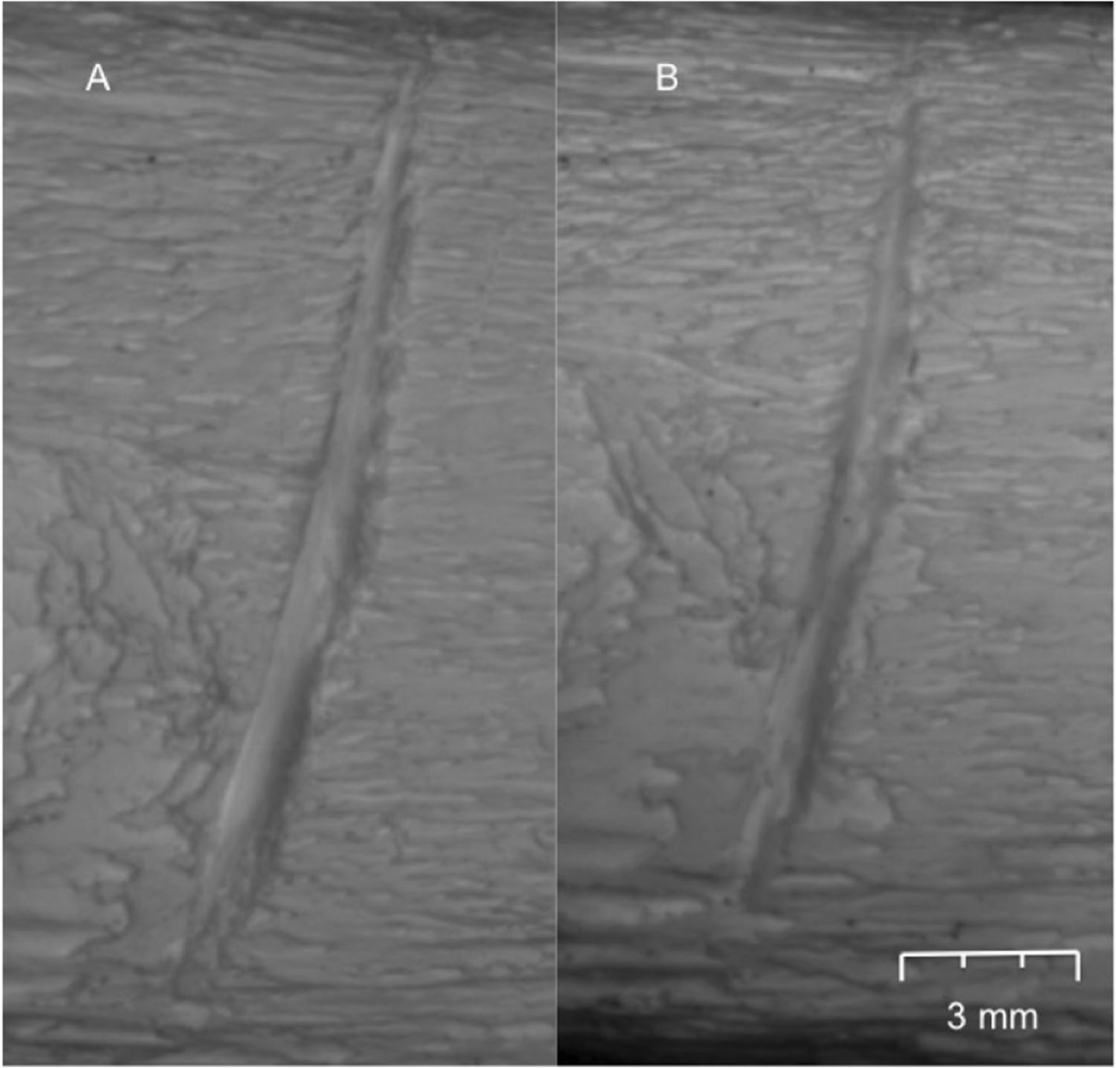
An example of the transformation from an elliptical to irregular kerf shape of a cut mark created by the coarse-serrated blade: **A** post-burning and **B** 4-week surface exposure

**Fig. 10:**
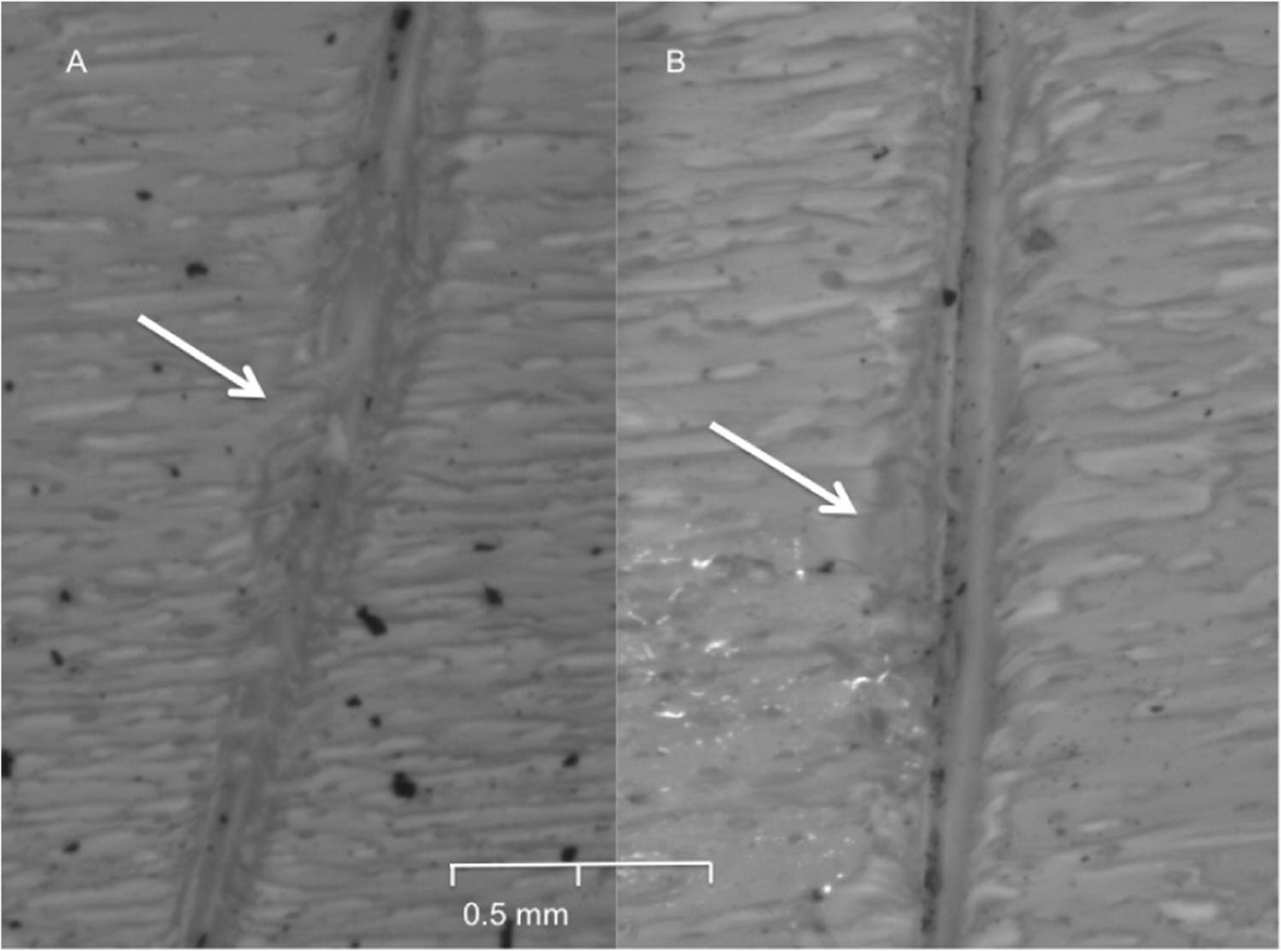
Raised kerf margin of a cut mark inflicted by coarse-serrated blade before (**A**) and after 1-month surface exposure (**B**) with marginal erosion in the winter; the white arrows indicate the same area of the kerf margin

In contrast, cut marks inflicted by the coarse-serrated blade underwent greater changes when compared with cut marks inflicted by the other knife blades. After 4 weeks of surface exposure, between 25 and 75% of elliptical shape marks tended to transform into an irregular shape (Fig. 9), whereas 16.7–33.3% of V-shaped cross-section cut marks changed to a U-shaped feature. Erosion of raised kerf margins and striations was also observed after environmental exposure (Fig. 10). Surface-deposited samples showed a greater degree of morphological alteration when compared with the buried samples, especially those samples exposed in the autumn and winter seasons. However, for all cut mark characteristics, there was no statistical significance between the post-burn and 2-week exposure groups. Nevertheless, morphological changes such as kerf shape and margin in the autumn group as well as kerf margin in the winter group of cut marks inflicted by coarse-serrated blade showed significant association (*p* < 0.05) after 4-week surface exposure (Table 5). Morphological alterations of cut marks inflicted by fine-serrated blade showed the same pattern as those inflicted by a coarse-serrated blade. However, with this latter blade, there was no statistical significance of morphological changes between post-burn and post-environmental exposure.

## Discussion

### Burnt bone fragmentation

It has been demonstrated in this study that the fragmented rate of burnt bones varied significantly depending on the duration of exposure, depositional environment, and weather. As demonstrated by other authors, weather conditions such as fluctuating temperature and rainfall can increase the degree of fragmentation [10]. In particular, the use of juvenile rather than adult pig bones in this experimentation may have contributed to a greater fragmentation due to the amount of organic material [11, 19]. In addition, if soft tissue was present, this may have influenced to a certain degree the degree of fragmentation due to the traction and shrinkage during burning of the of soft tissues surrounding the underlying bone [5, 20]. While this study focused solely on ribs, different bone types with different internal architectures could also undergo different degrees of fragmentation.

An increase in burnt bone fragmentation was to be expected over time [10, 13]. The burnt bones recovered after 4-week surface exposure revealed a higher degree of fragmentation compared to that recovered after 2-week surface exposure. The highest degree of fragmentation was observed in the autumn sample, evidencing a higher proportion of small- and medium-sized categories at the 4-week interval. Heavy rain and high precipitation, which were the most evident weather condition in the autumn, can increase the rate of fragmentation [10]. Likewise, the effect of heavy rainfall was observed during the first and fourth weeks of the spring and the fourth week of the summer, resulting in an increase in the rate of fragmentation. Nevertheless, it is not entirely understood how precipitation and heavy rainfall affect the bone structure [10]. Possible causes may be due to the penetration of water into any fissures or microcracks in the bone. In addition, when a wet burnt bone starts to dry, the loss of moisture may alter the pressure and strain of the bone structure, leading to further damage and fragmentation [10, 21].

A different trend of the survival of the summer samples compared with those of other seasonal samples was detected. The large fragments of burnt bone survived better after a 4-week surface exposure over the summertime. In addition, a decrease in the proportion of the medium- and small-sized categories reflects a slower rate of large bone fragmentation. The highest hours of sunshine in the summer cause vegetation to grow faster and cover burnt bones. The protective circumstance could protect fragile burnt bones against physical damage such as raindrop and strong wind, leading to a slower rate of fragmentation. In contrast, a high level of wind speed was observed during the autumn and winter, and this may have contributed to the loss of small-sized fragments in the winter in particular.

This study attempted to describe a correlation between the underground environment and fragmentation rate and pattern. A higher rate of burnt bone fragmentation was observed in the buried environment with high moisture content, especially in the spring and autumn. In contrast, the lowest level of soil moisture in the summer showed the lowest level of fragmentation. As previously discussed, an environment with high humidity increases the fragmentation rate because water can penetrate into microcracks and fissures in burnt bone tissue, weakening its structure [10]. In addition, with even mildly acidic soil (as in this study), there can be a reaction between the mineral component of the bone, soil, and water, resulting in the dissolution of the mineral and therefore the structural integrity of the bone. As the dissolution rate of the bone can increase with the water flow rate, this could well be a contributory factor in the increase in the fragmentation rate in the wetter periods [22, 23]. A correlation between soil pH and burnt bone fragmentation was not significant.

Regarding the pattern of fragmentation in the buried samples, a predominance of the medium-sized fragmentation category was detected for every season. As observed, surface-deposited burnt bones were more fragmented compared to buried burnt bones.

### Alterations of burnt bone weight

A reduction in bone mass is expected in burnt bone due to the loss of water and organic material [2, 4, 24, 25]. The literature reports a weight loss of 30–60% of the original weight in bone after heat exposure depending on associated factors such as temperature, duration, and conditions of a bone prior to burning [4, 24, 25]. Nevertheless, this study revealed a higher decrease than those in previous literature, with 71.1–77.7% of original weight loss after heat exposure. However, Gonçalves et al. (2013) used adult human remains cremated in a gas-fueled furnace [24], and thus, a comparison with different animal species and different age categories may be problematic.

This study showed a relationship between weather conditions and burnt bone weight. It is clear that a significant loss of bone weight was observed in the surface-deposited samples during the autumn and winter. These findings were similar to the rate of burnt bone fragmentation. A decrease in burnt bone mass might be the result of the loss of small bone fragments that could not be recovered and the loss of bone fragments by physical factors such as strong wind [10, 11, 13]. It is evident in the winter that the loss of bone weight was considerable after 4-week surface exposure. This result was associated with the smallest category of fragmentation.

The current study observed an association between the loss of bone weight and fragmentation in the buried samples. The results illustrate that the buried burnt bone weight of the spring and the autumn was stable. The lack of clear seasonal differences in the loss of bone weight with time indicates that any dissolution of the bone is not the dominant factor. A loss of burnt bone mass in burial conditions may be influenced by the problem of total recovery of very small bone fragments. Perhaps sieving of the soil would be an element to include, but it can also create further fragmentation.

### Survival of cut marks

An examination of heat-exposed bones, when combined with subsequent environmental exposure, is complex with many factors affecting how the burnt bones may be modified [14, 26]. Burnt skeletal remains and their traumatic lesions are subject to damage, fragmentation, and dispersion by the depositional environment and weather conditions [10, 27, 28]. Detailed knowledge of how the prevailing weather conditions and microenvironment have on burnt bones and their traumatic marks is crucial for our analysis of skeletal trauma in forensic cases.

Despite extensive fragmentation of the burnt bones, most of the cut marks on burnt bones were still identifiable after surface exposure. The same proportion was found in the spring and summer groups, with only 10% and 34% of the 2-week and 4-week surface groups respectively, unrecognizable (Fig. 7). These should be the result of the similar atmospheric phenomena in both seasons, with a low level of rainfall and slow wind speed. Nevertheless, considerable damage of the cut marks in the surface sample from the autumn and winter groups was observed. The autumn cut marks were less damaged in the first 2 weeks, with 33% compared to the 40% of the winter cut marks. After 4-week surface exposure, 60% of the winter cut marks had disappeared. The freezing environment during the winter season should be the main cause of this phenomenon. Ice crystals may form within pores in the bone tissue, and the volume expansion can cause the bone to crack and fragment [29, 30]. Though heat-exposed bone is dehydrated, Pokines et al. (2018) explained that a bone can re-uptake moisture within its porous structure as well as within the new fracture(s) [21]. Waterhouse (2013a) stated that freezing temperatures could hasten burnt bone fragmentation [10]. Tersigni (2007) used a scanning electron microscope to observe the consequence of the expansion of frozen water after bone was exposed to freezing temperatures for 3 weeks and found that microcracks originated among the Haversian systems [31]. This microscopic damage has the potential to develop into macroscopic features with prolonged exposure.

The highest rate of cut mark deterioration was observed in the 4-week surface autumn group, with 74% of the cut marks being unrecognizable. Heavy rain was detected during the last 2 weeks of the autumn period, which probably had an effect on burnt bone structure. Water molecules may increase the fragmentation rate by accessing the microfissures via the porous surface [10]. Furthermore, some matrix minerals may be dissolved [21]. A longer period of rainfall exposure therefore can produce much more damage to the burnt bone. Thus, the potential to recognize important features of a cut mark is considerably hindered in these scenarios.

### Dimensional and morphological changes of environment-exposure cut marks

In this study, a steady decrease in the dimension of most of the cut marks in the surface and buried burnt bone samples was observed. However, an increase in cut mark width was observed in the autumn and winter surface-deposited group. These changes were observed only in the cut marks inflicted by coarse-serrated and fine-serrated blades. It is possible to explain these phenomena by morphological erosion of the kerf margins. Even though raised kerf margins survived the burning process, their structures were fragile and vulnerable to taphonomic modifications (Fig. 10). After 4-week surface exposure, the cut marks inflicted by coarse-serrated and fine-serrated blades were likely to lose their margin regularity, with a significant loss of information about the type of knife used. Unlike the surface environment, the buried environment substantially reduced the rate of fragmentation resulting from the protection against physical damage and environmental fluctuation [22, 32]. Although burnt bone is likely to degrade in acidic soils [33], mildly acidic soil in this study was able to better preserve the buried burnt bone compared to that from the surface exposure [34]. Buried samples also showed less damage to kerf margins after 4-week exposure in the autumn and winter periods. Unlike the cut marks inflicted by a coarse-serrated and fine-serrated knife blade, those inflicted by a non-serrated blade in this study were still identified. Their morphological changes had few modifications after 4-week environmental exposure. It is thus expected that the cut marks inflicted by a non-serrated blade are well preserved and their individualization can be identified despite exposure to prolonged surface environment.

## Conclusions

This study has provided knowledge on the taphonomic factors that may influence the survival of cut marks in burnt bone in both buried and surface environments. These results contribute to our understanding of factors affecting bone fragmentation and cut mark analysis in forensic anthropology casework.

Environmental exposure resulted in fragmentation of all the rib bones, with the number of fragments increasing with time and being greater in those exposed to the surface than the buried bones. Importantly, it was seen that the fragmentation extended from heat-induced fractures, the cut marks having no noticeable effect on fragmentation, and consequently the majority (72.2%) were still identifiable. However, the degradation was far worse in the autumn and winter seasons than the other seasons.

Exposure resulted in changes to cut mark lengths and widths. The degree to which cut mark morphological characteristics changed increased from the non-serrated, to fine-serrated to coarse-serrated blade, with the latter being badly damaged and unexaminable.

The most destructive exposure period for this UK-based study was in the autumn suggesting heavy precipitation and wind as the driving factor. The mechanism is unclear, needing further study, but cyclical wet-dry periods and microcracks could induce internal strains within the bone. For buried samples, dissolution of the bone mineral due to the interaction of the mineral, soil, and water could be a contributory factor.

It is however clear that substantial further work is required. For a given location (fixed soil type), cut marks on different bone types and the effect of burning by fire would be the next future steps. In addition, the fine surface detail that is required for some cut mark characteristics can only be imaged by casting and using oblique lighting/scanning electron microscopy. Studies incorporating this level of detail are needed to assess the changes of the more subtle morphological cut mark features.

## Data Availability

The authors confirm that the data supporting the findings of this study are available within the article.
